# Baseline Prevalence of Trachoma in 21 Local Government Areas of Adamawa State, North East Nigeria

**DOI:** 10.1080/09286586.2021.2013899

**Published:** 2021-12-26

**Authors:** Mohammed Dantani Adamu, Aliyu Mohammed Jabo, Philomena Orji, Yaobi Zhang, Sunday Isiyaku, Nicholas Olobio, Nasiru Muhammad, Buwalky Barem, Rebecca Willis, Ana Bakhtiari, Cristina Jimenez, Anthony W. Solomon, Emma M. Harding-Esch, Caleb D. Mpyet

**Affiliations:** 1 Department of Ophthalmology, Usmanu Danfodiyo University, Sokoto, Nigeria; 2 Helen Keller International, Nigeria Country Office, Abuja, Nigeria; 3 Helen Keller International, Regional Office for Africa, Dakar, Senegal; 4 Sightsavers Nigeria Country Office, Kaduna, Nigeria; 5 Federal Ministry of Health, Abuja, Nigeria; 6 Ministry of Health, Yola, Nigeria; 7 International Trachoma Initiative, Task Force for Global Health, Decatur, Georgia, USA; 8 Sightsavers, Haywards Heath, UK; 9 Department of Control of Neglected Tropical Diseases, World Health Organization, Geneva, Switzerland; 10 Clinical Research Department, London School of Hygiene & Tropical Medicine, London, UK; 11 London Centre for Neglected Tropical Disease Research, London, UK; 12 Department of Ophthalmology, University of Jos, Jos, Nigeria

**Keywords:** Trachoma; Adamawa; Prevalence; WASH; Survey

## Abstract

**Purpose:**

To determine the prevalence of trachoma in each of the 21 local government areas (LGAs) of Adamawa State, Nigeria.

**Methods:**

A population-based cross-sectional survey was conducted in each of the 21 LGAs of Adamawa State between 2017 and 2019. With the support of Tropical Data (TD), surveys were planned and implemented in accordance with World Health Organization (WHO) recommendations. A two-stage cluster sampling technique was used in each LGA, 25 or 30 clusters were selected with a probability of selection proportionate to cluster size, and in each of these clusters, 25 or 30 households were enrolled for the survey. All residents aged 1 year and older within selected households were examined by TD-certified graders for trachomatous inflammation – follicular (TF) and trachomatous trichiasis (TT) using the WHO simplified grading scheme. Additionally, data were collected on household water and sanitation access.

**Results:**

All 21 LGAs had TF prevalence in 1–9-year-olds below 5%. The prevalence of TT unknown to the health system in people aged ≥15 years was ≥0.2% in three of the 21 LGAs. Access to improved water and sanitation facilities was <80% in the majority of the surveyed LGAs. Only 12 of the 21 LGAs had ≥50% household-level improved latrine access, and only Yola North had ≥80% household-level improved latrine access.

**Conclusion:**

There is no need for mass treatment with antibiotics for trachoma elimination purposes in any of these LGAs. There is a need for active TT case finding and provision of community-based TT surgical services in three LGAs. Furthermore, engagement with water and sanitation agencies is needed to augment access to improved water and sanitation facilities across the State; this will help to avoid the recrudescence of active trachoma in the State.

## Introduction

Trachoma is the leading infectious cause of blindness worldwide.^1^ In 1996, the World Health Organization (WHO) Alliance for the Global Elimination of Trachoma by 2020 (GET2020) set the year 2020 as the target date for the global elimination of trachoma as a public health problem^2^; this has recently been updated to 2030.^3^ The recommended intervention package to eliminate trachoma is the SAFE strategy (Surgery for trachomatous trichiasis (TT), antibiotics to clear infection, and promotion of Facial cleanliness and Environmental improvement to reduce transmission).^4,5^ The WHO recommends mapping of trachoma and implementation of elimination activities at the district level; for trachoma elimination purposes, this is defined as the normal unit of health administration with a population of 100,000–250,000 people.^6^ In Nigeria, this equates to a Local Government Area (LGA). The decision to implement the SAFE strategy and two of the three criteria to declare elimination of trachoma as a public health problem (prevalence of trachomatous inflammation – follicular (TF) <5% in 1–9-year-olds and prevalence of TT < 0.2% in ≥15-year-olds), are all based on district-level, population-based prevalence data.^5,7^

Adamawa State is located in the north-eastern zone of Nigeria; it has 21 LGAs and shares boundaries with Gombe, Taraba, and Borno States while on its eastern border lies the Far Northern and Adamaoua Regions of Cameroon. Trachoma is a notifiable disease in Adamawa State, although cases are seldom reported.^8^ Community-based surveys are needed to accurately estimate prevalence. Prior to this series of surveys, Adamawa State had not been mapped for trachoma, largely due to security challenges, and it did not have an existing trachoma programme. Surrounding administrative areas in both Nigeria and Cameroon had previously been mapped and documented to have trachoma requiring interventions.^9–12^

This study sought to determine the LGA-level prevalence of TF and TT in Adamawa so that the government and its partners could determine whether a trachoma elimination program would be required. Additionally, given the association between ocular *Chlamydia trachomatis* transmission intensity and (a) limited access to water for hygiene purposes^13^ and (b) increased availability of breeding sites for the human-faeces-breeding trachoma vector *Musca sorbens*,^14–16^ household-level access to water, sanitation and hygiene (WASH) facilities was also assessed.

## Materials and methods

From 2017 to 2019, we conducted LGA-level, baseline, and cross-sectional population-based prevalence surveys for trachoma in Adamawa State. Fourteen LGAs were surveyed in 2017 and seven in 2019. WHO protocol recommendations^17^ and standardised implementation guidelines from Tropical Data (TD) were followed, including TD’s standardised trachoma grading certification. Versions 1 (2017) and 2 (2019) of the TD training system^18,19^ were used. We implemented standard quality assurance and quality control measures for trachoma surveys that have been described in detail elsewhere.^20^

### Sample size calculation

As is customary for trachoma surveys, the TF prevalence in 1–9-year-olds was considered the primary outcome measure, and the number of households that should be included in each constituent survey was determined on the basis of the number needed to have reasonable confidence in enrolling and examining sufficient children to estimate TF prevalence with acceptable precision. TT prevalence in ≥15-year-olds was estimated amongst ≥15-year-olds examined in the same households.

The sample size of 1–9-year-olds for estimating the TF prevalence in each LGA was determined using the single-population-proportion-for-precision formula,^17,21^ whereby sample size (n) is equal to D_eff_ × NRIF × Z × (p(1 − p))/(d^2^). For our calculations, the design effect (D_eff_) was set as 2.65; the non-response inflation factor (NRIF) was set as 1.2; the z-score for the risk of alpha-error (Z) was 1.96; the expected proportion of children with TF (p) was 10% and the absolute precision required (d) was ±3%.^21^ The calculated target sample size was therefore 1225 children.

### Sampling technique

The evaluation unit in this survey series was the LGA. For each of the 21 LGAs, a two-stage cluster sampling strategy was used to select the study population. Villages were first-stage clusters. A list of the villages in each LGA was used as the sampling frame, from which 25 clusters (in 14 LGAs – for the 2017 surveys), and 30 clusters (in 7 LGAs – for the 2019 surveys) were selected using systematic sampling with a probability-proportional-to-village-size methodology.^5^ In 2017, the cluster number was determined based on each survey team being able to reliably survey 25 households in 1 day, and there being about 2 children aged 1–9 years in each rural household in northern Nigeria (1225/(25 × 2) = 24.5).^22^ In 2019, the number of households per day was expanded to 30, and the cluster number was increased to 30 to be more certain of meeting the sample size requirement for estimating both TF and TT prevalence.^17^ The second-stage clusters were households. These were selected using a compact segment sampling method. Briefly, villages were divided into segments that contained approximately 25 or 30 households (in 2017 and 2019, respectively) and one segment was selected by drawing lots. All the households in the selected segment were invited to take part in the survey.

The WHO simplified grading scheme, as set out in 1987,^23^ was used to grade trachoma. All graders were ophthalmic nurses who had participated in a TD grader qualifying workshop and passed both the slide-based and live patient inter-grader agreement tests with kappa statistics ≥0.70 for the diagnosis of TF.^18,19^ Data recorders also completed analogous TD processes of standardised training, testing, and certification.

Within each household, all the residents aged ≥1 year who had lived in the household for at least 6 months at the time of survey, including those absent at the time of the survey team’s visit, were enumerated and invited to be examined. Household members present at the time of the survey and willing to participate were examined for TT and TF. The teams returned at the end of the day to examine consenting persons who had been absent earlier.

Each grader used a 2.5 × magnifying loupe to examine participants under daylight illumination. In any eye graded as having TT, the grader also assessed whether trachomatous scarring (TS) was present.^24^ For the purposes of this paper, we defined TT as the presence of trichiasis in either the upper or lower eyelid, with or without TS. Those with TT were asked whether they had been offered and/or received management for their TT. We defined a case of TT unknown to the health system as someone with TT who had not yet been offered management or who could not remember being offered management in at least one eye. In 2019 surveys, follicle size guides^25^ were used to help graders consistently make a distinction between follicular inflammation that met or did not meet the definition of TF.^23^

### Data collection and data management

Data were collected, stored, and managed using the TD Android phone-based data collection app, and were encrypted and transferred in near real-time to TD servers for cleaning, analysis, and storage.

### Household-level data

GPS coordinates for each selected household were recorded. Additional data on water and sanitation facilities were collected by administering a questionnaire – adapted from the WHO/United Nations Children’s Fund (UNICEF) Joint Monitoring Program (JMP) for Water Supply and Sanitation household questionnaire^18,19,21^ – to the household head or their proxy. Household latrine and handwashing facilities (if present) were inspected to capture details of their type (latrines) or presence/absence of water (hand washing facility). Water sources and sanitation facilities were categorised as improved or unimproved, as per the WHO/UNICEF JMP definitions used for monitoring progress towards the Sustainable Development Goals.^26^ A household was defined as a compound head together with all individuals normally resident in the compound and eating from the same pot.

### Ethics

Survey protocols were approved in advance by the Ethics Committee of the Ministry of Health of Adamawa State, the National Health Research Ethics Committee of Nigeria (NHREC/01/01/2007) and the Observational/Interventions Research Ethics Committee of the London School of Hygiene & Tropical Medicine (16105). After field teams explained the examination protocol to each adult in an appropriate language, informed verbal consent for enrolment and examination was obtained. Verbal consent was agreed by the responsible ethics committees to be culturally appropriate in Adamawa, where adult literacy rates are low. Individuals aged ≥18 years consented for themselves; heads of households gave consent for the participation of minors (<18 years). Consent (or its refusal) was formally documented in the TD data collection app by the data recorder, who had a unique identification number. Individuals with active (inflammatory) trachoma were given two tubes of 1% tetracycline eye ointment, and they or their carer instructed to apply it to the lower conjunctival fornix of both eyes twice daily for 6 weeks; persons with TT were referred for eyelid surgery at the nearest facility to the village. Examiners cleaned their hands with an alcohol-based skin cleaning agent after the examination of each participant.

### Data analysis

Data were analysed in R (R Foundation for Statistical Computing, Vienna, Austria).^27^ Cluster-level TF prevalence was adjusted for age in one-year bands using the most recent census data.^28^ LGA-level TF prevalence estimates were generated as the mean of the adjusted cluster-level TF prevalence estimates. Similarly, cluster-level TT prevalence was adjusted for age and gender in five-year age bands using the most recent census data, and LGA-level TT prevalence generated as the mean of adjusted cluster-level prevalence estimates. Confidence intervals (CIs) were calculated by taking the 2.5th and 97.5th centiles of the distribution of prevalence estimates generated by repeatedly resampling cluster-level estimates over 10,000 iterations.

## Results

Surveys were conducted in 21 LGAs; in February 2017 (14 LGAs) and March 2019 (7 LGAs). A total of 76,096 people were examined; 54% were female. The ages of persons examined ranged from 1 to 100 years. A total of 35,731 children aged 1–9 years were enumerated, of whom 35,338 (99%) were examined, and 36,517 adults aged ≥15 years were enumerated, of whom 33,040 (90%) were examined. The age and gender distribution of the study population for all LGAs combined is shown in Table 1.

**Table 1.** Age and gender distribution of study participants surveyed for trachoma in 21 LGAs of Adamawa State, Nigeria, February 2017–March 2019.

Age (years)	Females	%	Males	%	Total	% of Total
1–10	18,333	24	19,290	25	37,623	49
11–20	6300	8	4458	6	10,758	14
21–30	6825	9	2328	3	9153	12
31–40	4779	6	3404	4	8183	11
41–50	2233	3	2765	4	4998	7
51–60	1223	2	1555	2	2778	4
61–70	741	1	879	1	1620	2
71–80	288	0.4	450	1	738	1
≥81	109	0.1	136	0.2	245	0.3
Total	40,831	54	35,265	46	76,096	100

### Prevalence of trachoma

**Figure 1.**
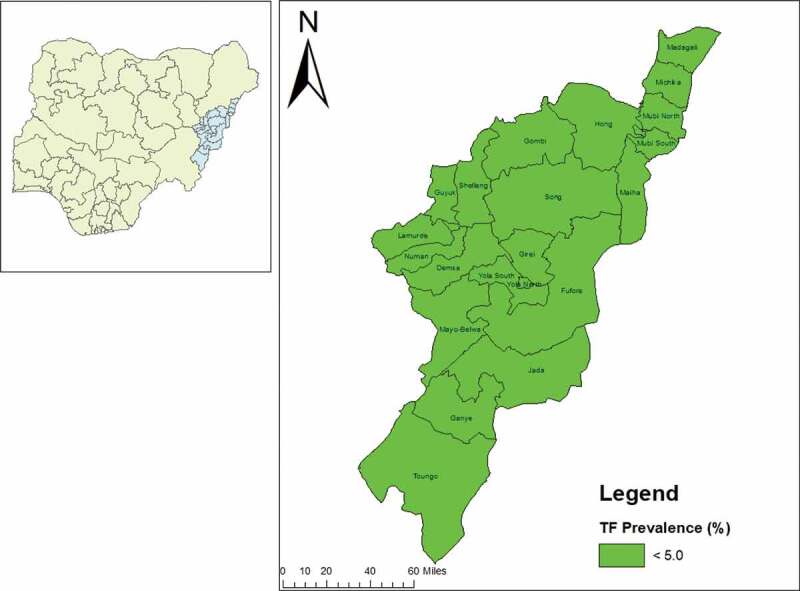
Prevalence of trachomatous inflammation-follicular (TF) in 1–9-year-olds, by Local Government Area, Adamawa State, Nigeria, February 2017–March 2019. The boundaries and names shown and the designations used on this map do not imply the expression of any opinion whatsoever on the part of the authors, or the institutions with which they are affiliated, concerning the legal status of any country, territory, city or area or of its authorities, or concerning the delimitation of its frontiers or boundaries.

**Figure 2.**
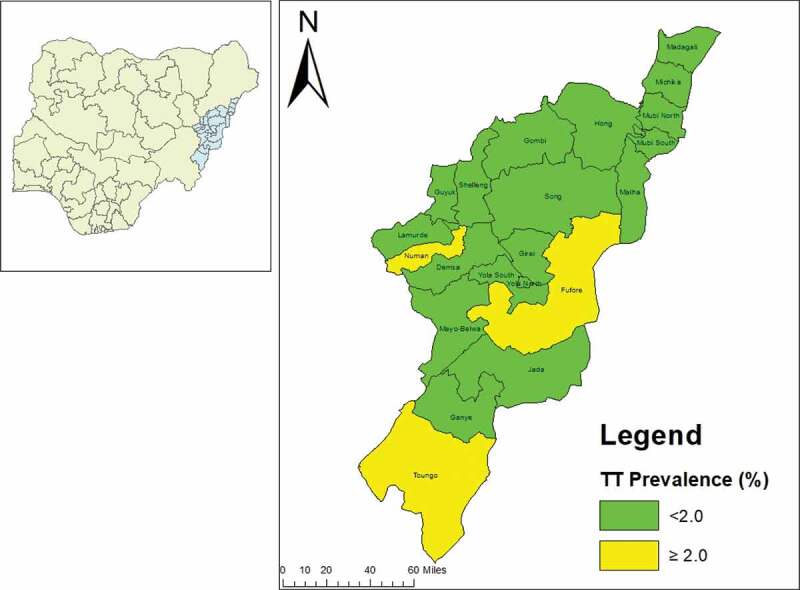
Prevalence of trachomatous trichiasis (TT) unknown to the health system in persons aged ≥15 years, by Local Government Area, Adamawa State, Nigeria, February 2017–March 2019. The boundaries and names shown and the designations used on this map do not imply the expression of any opinion whatsoever on the part of the authors, or the institutions with which they are affiliated, concerning the legal status of any country, territory, city or area or of its authorities, or concerning the delimitation of its frontiers or boundaries.

The prevalence of TF in children aged 1–9 years ranged from 0.4% in Maiha LGA to 3.6% in Yola North LGA. There was no LGA with a TF prevalence of ≥5% (Table 2, Figure 1). The prevalence of TT unknown to the health system in persons aged ≥15 years was <0.2% in 18 LGAs. In Fufore, Numan, and Toungo LGAs, the prevalence of TT unknown to the health system was ≥0.2% (Table 3 and Figure 2). The backlog of TT in Adamawa State, which has an estimated total population of 3,168,101^28^, was calculated^29^ to be 2,008 (Table 3).

**Table 2.** Local government area-level prevalence of trachomatous inflammation-follicular (TF) in 1–9-year-olds, Adamawa State, Nigeria, February 2017–March 2019.

Local Government Area			1–9-year-olds		Prevalence (%)	(95% CI)
	Year of survey	Number of clusters	Enumerated	Examined		
Demsa	2017	25	1786	1770	1.2	(0.3–2.8)
Fufore	2017	25	1835	1814	1.2	(0.4–2.3)
Ganye	2017	25	1761	1741	0.9	(0.4–1.5)
Girei	2017	25	1627	1601	0.5	(0.1–0.9)
Gombi	2017	25	1869	1856	0.8	(0.4–1.1)
Guyuk	2017	25	1797	1776	0.7	(0.2–1.2)
Jada	2017	25	1712	1693	0.5	(0.1–1.1)
Lamurde	2017	25	1573	1557	0.6	(0.3–1.0)
Mayo Belwa	2017	25	1662	1653	0.8	(0.4–1.4)
Numan	2017	25	1604	1591	0.5	(0.1–1.1)
Shelleng	2017	25	1790	1777	1.6	(0.9–2.4)
Song	2017	25	1919	1885	0.9	(0.3–1.8)
Yola North	2017	25	1254	1224	3.6	(1.3–5.7)
Yola South	2017	25	1502	1479	1.3	(0.3–2.2)
Hong	2019	30	1766	1746	0.6	(0.2–0.9)
Madagali	2019	30	1381	1365	0.5	(0.1–1.1)
Maiha	2019	30	2041	2020	0.4	(0.1–0.7)
Michika	2019	30	1619	1611	0.6	(0.1–1.3)
Mubi North	2019	30	1722	1703	1.0	(0.1–2.7)
Mubi South	2019	30	1918	1900	0.8	(0.2–1.3)
Toungo	2019	30	1593	1576	0.8	(0.3–1.5)

**Table 3.** Prevalence of trachomatous trichiasis (TT) unknown to the health system and TT backlog in 21 LGAs of Adamawa State, Nigeria, February 2017–March 2019.

Local Government Area	Estimated total population of persons aged ≥15 years^a^	≥15-year-olds		Prevalence^b^ (%)	(95% CI)	Estimated TT backlog^c^
		Enumerated	Examined			
Demsa	100,941	1599	1425	0.20^d^	(0.06–0.38)	202
Fufore	116,081	1786	1571	0.29	(0.00–0.85)	337
Ganye	91,889	1560	1389	0.09	(0.00–0.21)	83
Girei	72,797	1625	1430	0.09	(0.00–0.17)	66
Gombi	82,000	1374	1209	0.09	(0.00–0.18)	74
Guyuk	99,560	1434	1244	0.10	(0.00–0.22)	100
Jada	94,345	1496	1345	0.09	(0.00–0.21)	85
Lamurde	63,170	1329	1132	0.19	(0.06–0.36)	120
Mayo Belwa	85,752	1404	1239	0.07	(0.00–0.20)	60
Numan	50,805	1548	1402	0.21	(0.05–0.43)	107
Shelleng	83,479	1314	1169	0.16	(0.00–0.46)	134
Song	107,910	1383	1238	0.02	(0.00–0.06)	22
Yola North	111,018	1623	1442	0.06	(0.00–0.17)	67
Yola South	108,980	1534	1303	0.07	(0.00–0.21)	76
Hong	94,711	2424	2221	0.04	(0.01–0.10)	38
Madagali	75,503	1997	1885	0.08	(0.00–0.18)	60
Maiha	62,280	2215	2095	0.09	(0.00–0.23)	56
Michika	86,969	2289	2154	0.08	(0.00–0.20)	70
Mubi North	84,600	2185	2037	0.10	(0.02–0.23)	85
Mubi South	72,205	2,243	2,095	0.12	(0.00–0.23)	87
Toungo	29,142	2,155	2,015	0.27	(0.11–0.48)	79
**Total (21 LGAs)**	**1,774,137**					**2008**

^a^Based on data from the 2006 census^28^

^b^Prevalence of TT unknown to the health system are displayed to two decimal places in order to provide clarity on whether or not the best estimate of prevalence was above or below the elimination threshold of 0.2% in adults ≥ 15 years

^c^TT backlog was calculated as per methods previously described^29^

^d^The data round to 0.20, but the prevalence estimate is < 0.20%

### Water and sanitation coverage

The proportion of households with access to improved drinking water sources was the lowest in Guyuk LGA (35%) and highest in Hong LGA (87%) (Table 4). Only two of the 21 LGAs had ≥80% of the households with access to an improved drinking water source. Drinking water access in the household or within 30 minutes of the household (regardless of status of the source as improved or unimproved) ranged from 59% in Michika to 89% in Madagali. Similarly, there was poor access to improved latrines across the State, with only 12 of 21 LGAs having ≥50% household-level improved latrine access and only 1 LGA having ≥80% household-level improved latrine access (Table 4). Household–level access to improved sanitation was lowest in Song (29%) and highest in Yola North (95%).

**Table 4.** Household access to improved drinking water source and improved latrines, by local government area (LGA), Adamawa State, Nigeria, February 2017–March 2019.

LGA	Number of households surveyed	Number of households with improved drinking water source N (%)	Number of households with drinking water source within 30 minutes return journey N (%)	Number of households with improved latrine N (%)
Demsa	622	325 (52)	529 (85)	228 (37)
Fufore	626	406 (65)	503 (80)	409 (65)
Ganye	626	317 (51)	481 (77)	411 (66)
Girei	625	468 (75)	535 (86)	395 (63)
Gombi	627	318 (51)	501 (80)	323 (52)
Guyuk	628	218 (35)	495 (79)	214 (34)
Jada	626	319 (51)	462 (74)	301 (48)
Lamurde	624	360 (58)	502 (80)	189 (30)
Mayo Belwa	625	264 (42)	491 (79)	256 (41)
Numan	626	265 (42)	457 (73)	261 (42)
Shelleng	625	303 (48)	551 (88)	277 (44)
Song	624	273 (44)	517 (83)	181 (29)
Yola North	621	325 (52)	548 (88)	591 (95)
Yola South	612	313 (51)	528 (86)	438 (72)
Hong	899	783 (87)	663 (74)	649 (72)
Madagali	896	649 (72)	793 (89)	600 (67)
Maiha	899	710 (79)	675 (75)	646 (72)
Michika	900	732 (81)	530 (59)	603 (67)
Mubi North	895	394 (44)	589 (66)	710 (79)
Mubi South	897	450 (50)	613 (68)	698 (78)
Toungo	898	654 (73)	589 (66)	434 (48)

## Discussion

This study suggests that TF is below the target for elimination as a public health problem in all LGAs in Adamawa State. According to current guidelines, no LGA in the State requires mass drug administration (MDA) of antibiotics for trachoma elimination.^5^ This is comparable to findings reported in neighbouring Gombe State,^10^ where none of the 11 LGAs surveyed required antibiotic MDA; Taraba State,^9^ where only one out of 13 surveyed LGAs required antibiotic MDA; and Northern Cameroon,^11^ where only 3 of 15 surveyed health districts required antibiotic MDA for trachoma elimination purposes. However, Madagali LGA does border some suspected endemic LGAs in Borno State, in addition to some previously trachoma-endemic districts in Cameroon, and therefore cross-border partnership is important to maintain these LGAs below the elimination prevalence threshold.^30^

To reduce the possibility of active trachoma recrudescence and maintain the low TF prevalence seen in this survey series, access to water and sanitation should be sustained and improved upon in all of Adamawa’s LGAs. In these surveys, inadequate access to improved water and sanitation facilities was recorded. The exception to this was Yola North, where 95% of households had access to improved sanitation. Community awareness should be raised on the health benefits of using improved sanitation; higher levels of access are associated with lower risk of trachoma.^15,31–33^ Water and sanitation agencies and other partners should engage with community leaders on this issue and determine ways to sustainably improve access.^34^ Such improvements would be likely to have substantial health benefits beyond trachoma^35^ and contribute towards the attainment of Sustainable Development Goals three (to improve global health and well-being) and six (to provide universal clean water and improved sanitation facilities).^36,37^

The prevalence of TT unknown to the health system was above the target for elimination of trachoma as a public health problem in Fufore, Numan, and Toungo LGAs. This finding is intriguing, given the low TF prevalence. Possible explanations could be due to the inclusion of lower eyelid trichiasis in the TT definition or that these present-day TT prevalences reflect past high TF prevalences, which have since been reduced to below elimination thresholds as a result of MDA. Even though only a few hundred people are in need of TT surgery in each one, the prevalence of TT meets the definition of a public health problem in these LGAs. It is therefore important for Adamawa State to undertake active TT case finding, train, and equip TT surgeons and deploy them to provide community-based services for these LGAs. These services should be durable: assuming that the trichiasis is due to trachoma, it is likely that people will continue to develop incident TT for some years.

This study was undertaken in an area that, like neighbouring Borno State,^38^ has faced security challenges for over a decade. During the period of the survey, several modifications and safety measures were undertaken; (i) In the second phase of the survey (2019) involving seven LGAs, the number of selected clusters was increased from 25 to 30 as the number of children encountered in the initial LGAs was too low. (ii) In some instances, no more than five villages had to be changed (another random selection of villages from a predefined list) as preselected villages came under attack by insurgents just as the survey team was due to deploy. To circumvent these security challenges and complete the surveys, the survey coordinator had to adopt a fluid itinerary that changed according to the security situation; survey teams were trained on local security conditions; satellite phones were carried; and a dedicated Security and Safety Officer was placed in charge of gathering and disseminating security-related information. We also worked with residents who advised field teams on the safest routes to take to selected villages and used local vehicles to avoid being marked out as visitors.

There were limitations to this study. Reliance on self-reporting for determining access to water could have led to inaccuracies, although direct observation was employed where possible to minimise this. Further, estimates of TT surgical backlog from survey data like these are acknowledged to be inaccurate because of the relatively small number of people examined in the age groups most at risk of TT. It is also worth noting that upper and lower eyelid trichiasis were not recorded separately during these surveys, precluding an analysis of the results in line with the new definition of TT agreed at the 4^th^ Global Scientific Meeting on Trachoma, November 2018.^39,40^

## Conclusion

These surveys show that in Adamawa State, prevalence of trachoma is generally low. No MDA intervention is required, and active TT case finding and other targeted surgical interventions are warranted in only three of the 21 surveyed LGAs. Behavioural practices that encourage facial cleanliness and infrastructure improvements in water access and human waste management should continue to guard against an increase in active trachoma prevalence in the future.
